# HP1β carries an acidic linker domain and requires H3K9me3 for phase separation

**DOI:** 10.1080/19491034.2021.1889858

**Published:** 2021-03-04

**Authors:** Weihua Qin, Andreas Stengl, Enes Ugur, Susanne Leidescher, Joel Ryan, M. Cristina Cardoso, Heinrich Leonhardt

**Affiliations:** a Center for Molecular Biosystems (BioSysM), Faculty of Biology, Ludwig-Maximilians-Universität München, Munich, Germany; b Department of Proteomics and Signal Transduction, Max Planck Institute for Biochemistry, Martinsried, Germany; c Cell Biology and Epigenetics, Department of Biology, Technical University of Darmstadt, Darmstadt, Germany

**Keywords:** Phase separation, chromatin structure, heterochromatin, histone posttranslational modification, heterochromatin binding protein HP1

## Abstract

Liquid-liquid phase separation (LLPS) mediated formation of membraneless organelles has been proposed to coordinate biological processes in space and time. Previously, the formation of phase-separated droplets was described as a unique property of HP1α. Here, we demonstrate that the positive net charge of the intrinsically disordered hinge region (IDR-H) of HP1 proteins is critical for phase separation and that the exchange of four acidic amino acids is sufficient to confer LLPS properties to HP1β. Surprisingly, the addition of mono-nucleosomes promoted H3K9me3-dependent LLPS of HP1β which could be specifically disrupted with methylated but not acetylated H3K9 peptides. HP1β mutants defective in H3K9me3 binding were less efficient in phase separation*in vitro *and failed to accumulate at heterochromatin *in vivo*. We propose that multivalent interactions of HP1β with H3K9me3-modified nucleosomes via its chromodomain and dimerization via its chromoshadow domain enable phase separation and contribute to the formation of heterochromatin compartments *in vivo*.

## Introduction

Liquid-liquid phase separation (LLPS) has recently emerged as a novel form of the cellular organization [1–4]. In addition to canonical membrane-bound organelles, phase separation forms membraneless organelles within cells to compartmentalize complex biological reactions in space and time. The formation of membraneless organelles is driven by intrinsically disordered proteins or disordered protein regions (IDR) [5,6]. Those proteins or protein domains are characterized by a low content of hydrophobic amino acids, biased amino acid composition, and low sequence complexity [5,7–10]. The cellular abundance of disordered proteins is tightly regulated and mutations in those proteins or changes in their cellular abundance are often associated with disease [11,12].

Heterochromatin binding protein HP1 is a non-histone chromosome binding protein and has a function in nuclear organization, chromosome segregation, telomere maintenance, DNA repair, and gene silencing [13,14]. In mammals, there are three homologs of HP1, termed HP1α, HP1β, and HP1γ, encoded by the genes *Cbx5, Cbx1,* and *Cbx3*, respectively. HP1 homologs have two conserved functional domains, an N-terminal chromodomain (CD) and a C-terminal chromoshadow domain (CSD), linked by a hinge region. The CD domain mediates recognition of di- and trimethylated K9 of histone H3 (H3K9me2 and H3K9me3) [15–17], while the CSD domain is responsible for interaction with other proteins and also mediates homo- and hetero-dimerization [18,19]. The intrinsically disordered regions and posttranslational modifications are likely responsible for the unique functions of HP1 homologs. Recent studies testing the capacity of HP1 to induce phase separation revealed that only HP1α formed phase-separated droplets [20–22]. This phase separation is initiated through intermolecular interaction of the phosphorylated N-terminus with the hinge region and correlates with the formation of heterochromatin and chromocenters in the nucleus. Although HP1β also predominantly accumulates at pericentromeric heterochromatin (chromocenters), it does not form phase-separated droplets under the conditions described for HP1α. It is though not clear how much LLPS mechanisms contribute to heterochromatin formation and clustering and, indeed, a model polymer-polymer/liquid-phase separation (PPPS or PLPS) has been recently proposed [23,24].

Chromatin organization undergoes dramatic changes during mammalian cell differentiation and proliferation. In proliferating cells, heterochromatin clusters (chromocenters) are disrupted during mitosis as they contain clustered centromeric and pericentromeric DNA from several chromosomes and then fuse again throughout interphase reaching the highest clustering in G2 and in terminally differentiated post-mitotic cells [25]. This fusion of chromocenters *in vivo* resembles the formation of phase-separated droplets *in vitro* and depends on the presence and concentration of heterochromatin proteins like HP1α and MeCP2 [20,22,25]. At the transition from pluripotent to differentiated stages, heterochromatin foci become more clustered and spherical [25,26], which correlates with lower exchange rates of chromatin proteins. HP1 proteins form homo- or hetero-dimers and have often been considered to play a rather equivalent role in heterochromatin organization. However, several lines of evidence suggest that the different HP1 proteins have specific functions in heterochromatin organization. For example, it has been shown that HP1α plays an important role in heterochromatin organization, while HP1β functionally associates with H4K20me3 [27,28]. HP1β has been suggested to act as a bridge linking H3K9me3 enriched condensed chromatin [29]. In addition, HP1α and HP1β likely play distinct roles during early embryo development, as they show different expression patterns [30].

To dissect functional differences of HP1 homologs in phase separation and chromatin organization, we compared the amino acid composition of HP1 proteins at disordered regions. We found that the charge of the IDR-H is a distinctive feature of HP1 homologs and plays a decisive role in LLPS and that HP1β undergoes phase separation in a histone H3K9me3 dependent manner. Hence, an HP1β mutant defective in H3K9me3 binding was deficient in phase separation and showed faster binding kinetics *in vivo*.

## Materials and methods

### Cell culture and transfection

Mouse E14 ESCs, cells were cultured in gelatinized flasks in DMEM supplemented with 16% fetal calf serum, 0.1 mM β-mercaptoethanol (ThermoFisher Scientific, Invitrogen), 2 mM L-glutamine, 1× MEM non-essential amino acids, 100 U/ml penicillin, 100 µg/ml streptomycin (Sigma-Aldrich, Germany), 2i (1 μM PD032591 and 3 μM CHIR99021 (Axon Medchem, Netherlands) and 1000 U/ml recombinant leukemia inhibitory factor LIF (Millipore). Human embryonic kidney (HEK) 293 T cells were cultured in DMEM supplemented with 10% fetal calf serum and 50 µg/ml gentamycin (PAA).

Mouse ESCs were transfected with Lipofectamine 3000 Reagent (ThermoFisher Scientific, Invitrogen) according to the manufacturer’s instructions.

### CRISPR/Cas-mediated gene editing and generation of stable cell lines

For the generation of GFP-HP1β WT and KW cell lines, the MINtag strategy was used as described previously [31]. In brief, HP1β specific gRNA was cloned into a vector expressing GFP and SpCas9 (px458: F. Zhang Lab). Mouse ESCs were transfected with the Cas9-gRNA vector and a 200 nt donor oligo coding for the MINtag. Two days after transfection, GFP positive cells were separated using FACS (Becton Dickinson, Germany) and plated at clonal density (2000 cells per p100 plate). After one-week, single clones were picked manually and transferred into two 96-well plates. Cell lysis in 96-well plates, PCR on lysates, and restriction digest were performed. To generate WT and KW GFP-HP1β cell lines, we used our MIN-tagged HP1β mESCs and inserted the WT or the KW GFP-HP1β coding sequence into the N-terminus of the endogenous *HP1β*^attP/attP^ locus by Bxb1 mediated recombination (Figure 5d). PCR primers for screening are as follows:

HP1β-ext F: 5ʹ-GATTTCCCTGGGCTCCTCAC-3ʹ

HP1β-ext R: 5ʹ-ATGCCCATCACAGAACTGCT-3ʹ

AttL-F: 5ʹ- CCGGCTTGTCGACGACG-3ʹ.

### Protein purification, histone, and mononucleosome isolation

HP1 cDNA was cloned into a pET28a (+) expression vector (Merck KGaA, Novagen), mutants were made using overlap extension PCR, and proteins were subsequently expressed in *E. coli*. For protein purification, BL21 cells were grown to OD 0.6–0.8 at 37°C, then IPTG was added to a final concentration of 0.5 mM and cultures were incubated at 18°C overnight. Harvested cells were resuspended in lysis buffer (20 mM Tris-HCl pH 8.2, 250 mM NaCl, 20 mM Imidazole, 3 mM β-Mercaptoethanol, 1 mM PMSF, 25 µg/ml DNase I and 100 µg/ml Lysozyme) and incubated at 4°C under constant rotation for 1–2 h. Following sonication, cell debris was removed by centrifugation at 20,000 x g for 30 min at 4°C. Clarified lysate was injected into an ÄKTA Purifier system (GE Life Sciences, Germany) equipped with a Ni-NTA column and His-tagged proteins were finally eluted in elution buffer (20 mM Tris/HCl pH 8.2, 250 mM NaCl, 500 mM Imidazole, and 3 mM β–Mercaptoethanol). The fractions with the highest purity were mixed and concentrated to about 1 µg/µl using Amicon® Ultra 4 mL centrifugal filter (Merck, Germany) in the buffer (20 mM HEPES pH 7.2, 200 mM KCl, 1 mM DTT, 10% glycerol) before flash freezing in liquid nitrogen. Protein concentrations were measured with the Pierce™ 660 nm protein assay kit (ThermoFisher Scientific) according to the manual.

Histone isolation was conducted as previously described with minor changes of the protocol [33]. In brief, 15 p100 plates of HEK293T cells were harvested and cell pellets were resuspended in a hypotonic buffer (10 mM Tris-HCl pH 8, 10 mM KCl, 1.5 mM MgCl_2_, 1 mM DTT, and 1x Protease Inhibitor, 2 mM PMSF). To obtain pure nuclei, cells were disrupted using a homogenizer and nuclei were subsequently incubated in a chromatin dissociation buffer (10 Tris-HCl pH 8.0, 20 mM EDTA, and 400 mM NaCl) for 30 min on ice. This chromatin dissociation step was repeated 4x. Afterward, nuclei were resuspended in 0.4 N H_2_SO_4_ and incubated on a rotator at 4°C overnight. After centrifugation, histones in the supernatant were transferred into a fresh reaction tube and precipitated using 33% trichloroacetic acid (TCA). After washing 3x with cold acetone, histones were dissolved in H_2_O and centrifuged at 2000 rpm for 5 min to remove precipitates. Histone concentrations were measured using the Pierce™ 660 nm protein assay kit.

For isolation of mononucleosomes, 3 × 10^7^ HEK293T cells were resuspended in 1 ml of hypotonic buffer containing 0.1% Triton-X 100, homogenized with 20 strokes in a Glass Teflon homogenizer and centrifuged at 1000 x g at 4°C to obtain intact nuclei. Nuclei were then resuspended in 800 µl of MNase digestion buffer (10 mM Tris-HCl, pH 7.4, 10 mM NaCl, 3 mM CaCl_2_, 0.1% NP-40, and protease inhibitors) supplemented with 40 U/ml MNase and incubated at 37°C for 5 min. The digestion was inactivated by a 5× stop buffer containing 10 mM Tris-HCl, pH7.4, 710 mM NaCl, and 7.5 mM EDTA. Mononucleosome extracts were cleared by centrifugation at 2 000 x g for 15 min at 4°C and the quality of the preparation was determined on an agarose gel after isolating DNA from the mononucleosome extracts.

### In vitro *droplet assays*

For the droplet assay, proteins were concentrated to ~10 µg/µl using Amicon® Ultra 4 mL centrifugal filter (Merck, Germany). After the concentration step, the buffer was exchanged to 20 mM HEPES pH 7.2, 75 mM KCl, 1 mM DTT with Zeba™ Spin Desalting Columns, and 1.4 nmol of HP1β were mixed with 1.4 nmol of histones in a total of 30 µl buffer at 4°C. 20 µl of the turbid solution was imaged in a 15 µ-Slide 18 Well ibidi chamber. Differential interference contrast (DIC) images were acquired on a DeltaVision Personal widefield microscope (GE Life Sciences) equipped with a 60 × 1.42 NA objective (Olympus), LED epi-illumination, and a CoolSnap ES2 camera (Photometrics).

For the spin-down assay, 30 µl of the turbid solution was spun down at 2000 rpm for 5 min and 29 µl of supernatant was transferred into a Protein LoBind Tube (Eppendorf). The supernatant and droplets were boiled in 250 µl Laemmli loading buffer at 95°C for 10 min. 5 µl of supernatant and droplets were loaded into an SDS-PAGE gel followed by either detection using coomassie staining or western blot analysis.

For HP1β phase separation with mononucleosome extracts, 28 µl of extract were incubated with 30 µg of HP1β in 30 µl solution for 5 min and spun down at 12,000 rpm for 5 min. 29 µl of supernatant was transferred into a Protein LoBind Tube and both supernatant and droplets were boiled in 40 µl Laemmli loading buffers at 95°C for 10 min. 20 µl of supernatant and droplets were again loaded into an SDS-PAGE gel followed by either detection using Coomassie staining or western blot analysis.

For comparison of histones and H3 peptides in HP1β phase separation, H3 peptides (aa 1–20) carrying H3K9me3 and biotinylated at the C-terminus were purchased from PSL GmbH, Heidelberg.

For the peptide competition assay, 25 µM of HP1β was incubated for 1 h with C-terminal TAMRA labeled histone H3 peptides (aa 1–20), containing H3K9me3, H3K9me1 or H3K9ac (PSL GmbH, Heidelberg) in a ratio of 1:5 or 1:50 in 30 µl buffers in Protein LoBind Tubes at 4°C. Then, 25 µM of histones were added to the solution and incubated at 4°C for 3 min. Droplets were separated by centrifugation at 2000 rpm for 5 min and 29 µl of supernatant were transferred into a new microfuge tube. Supernatant and droplets were boiled in 200 µl Laemmli loading buffers at 95°C for 10 min and 6 µl of each sample was loaded into an SDS-PAGE gel for detection by coomassie staining and TAMRA fluorescence.

### Analytic ion-exchange chromatography (IEX) and size-exclusion chromatography (SEC)

The surface charge of HP1 variants was analyzed by anion exchange chromatography. 50 µg HP1α, HP1β, or HP1γ were diluted in 500 µL buffer A (20 mM Tris-HCl, pH 8.0) and loaded on a 1 mL Resource Q column at room temperature and 4 ml/min flow rate using a Äkta Pure FPLC system. Samples were eluted with a linear gradient over 20 column volumes (CV) to 50% buffer B (20 mM Tris-HCl, 1 M NaCl, pH 8.0) followed by 10 CV 100% buffer B. Absorption at 280 nm was recorded.

250 µg of extracted histones were diluted in 50 µL SEC running buffers (20 mM Tris-HCl, 300 mM NaCl, pH 7.4). The sample was separated on an equilibrated Superdex 200 Increase 10/300 GL column at room temperature and 0.75 ml/min flow rate using a Äkta Pure FPLC system. Absorption at 280 nm was recorded. For size comparison, a protein gel filtration marker mix (Sigma-Aldrich) including carbonic anhydrase (29 kDa), bovine serum albumin (66 kDa), alcohol dehydrogenase (150 kDa), beta-amylase (200 kDa), apoferritin (443 kDa), thyroglobulin (669 kDa) was analyzed under identical conditions.

### Antibodies for western blot analysis

Primary antibodies used for western blot, including the polyclonal rabbit anti-H3 (Cat # ab1791), anti-H3K9me3 (Cat # ab8898), and anti-HP1β (Cat #10478) antibodies, were purchased from Abcam and the secondary antibody, anti-rabbit-IgG AF647 (Cat # A32733), from Invitrogen. The primary mouse monoclonal anti-H1 antibody (H-2) was purchased from Santa Cruz Biotechnology (Cat # sc-393358) and the secondary antibodies, anti-mouse IgG-HRP (Cat # A9044), and anti-rabbit IgG-HRP (Cat # A6154) were purchased from Sigma-Aldrich.

### Immunofluorescence staining

mESCs were washed with phosphate-buffered saline (PBS) and fixed with 3.7% formaldehyde in PBS, permeabilized with 0.5% Triton X-100 in PBS, and then blocked with 3% BSA. Cells were then incubated with a rabbit polyclonal anti-H3K9me3 antibody (Abcam, Cat # ab8898) or a rabbit polyclonal anti-HP1β antibody (Abcam, Cat #10478) for 1 hour at RT. After washing, cells were incubated with Alexa594-conjugated donkey anti-rabbit IgG secondary antibody (Invitrogen, Cat # A21207) for H3K9me3 and Alexa488-conjugated goat anti-rabbit IgG (Invitrogen, Cat # A11034) for HP1β 1 hour at RT. Nuclei were stained with 4ʹ,6-diamidino-2-phenylindole (DAPI) and mounted on coverslips with Vectashield (Vector Laboratories). Images were taken using an SP5 Leica confocal microscope equipped with Plan Apo 63x/1.4 NA oil immersion objective and lasers with excitation lines: 405 nm for DAPI, 488 nm for HP1β and GFP-HP1β, and 594 nm for H3K9me3.

### FRAP analysis

FRAP experiments were performed on an UltraVIEW VoX spinning disc microscope with an integrated FRAP PhotoKinesis accessory (PerkinElmer) assembled onto an Axio Observer D1 inverted stand (Zeiss) and using a 100×/1.4 NA Plan-Apochromat oil immersion objective. The microscope was equipped with a heated environmental chamber set to 37°C. Fluorophores were excited with a 488 nm solid-state diode laser line. Confocal image series were typically recorded with 16-bit image depth, a frame size of 512 × 512 or 256 × 256 pixels, and a pixel size of 69 nm. The bleach regions, typically with a diameter of 2 μm, were manually chosen to cover chromocenters. Photobleaching was performed using one iteration with the acousto-optical tunable filter (AOTF) of the 488 nm laser line set to 100% transmission. Twenty prebleach images were acquired at maximum speed, then 60 post-bleach frames were recorded at maximum speed followed by 30 frames at a rate of 3 s per frame. Data correction, normalization, and quantitative evaluations were performed by processing with ImageJ (http://rsb.info.nih.gov/ij/) followed by calculations in Excel. For normalization, the average intensity of five prebleach images was used.

## Results

### HP1β differs from HP1α and HP1γ in that it contains an acidic linker domain

**Figure 1.**
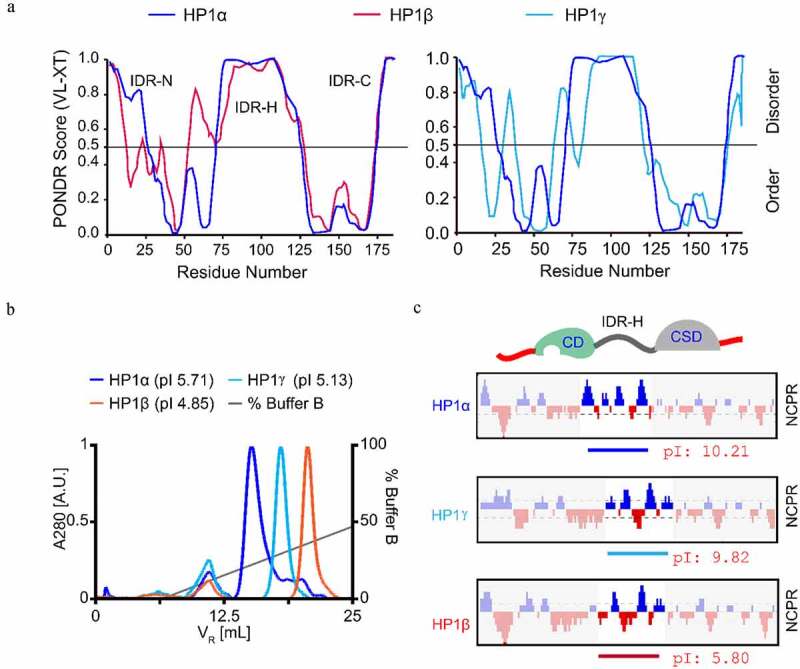
HP1β differs from HP1α and HP1γ in that it contains an acidic linker domain. (a) Comparison of order/disorder prediction of HP1 homologs by the PONDR algorithm, a website tool (http://www.pondr.com/). VLXT scores are shown on the y-axis, amino acid positions are shown on the x-axis. (b) Ion exchange chromatography analysis of HP1 proteins. 50 µg of HP1 proteins were diluted in 500 µL buffer B (20 mM Tris-HCl, pH 8.0) and loaded on a 1 mL Resource Q column and analyzed by using a Äkta Pure FPLC system. (c) Net charge distribution per residue (NCPR) of HP1 proteins (CIDER, pappulab.wustl.edu). Negatively charged amino acids are marked in red, positively charged amino acids in blue. The pI of IDR-H in HP1 proteins is indicated

Although the three HP1 homologs are very similar in their overall structure, only HP1α was reported to undergo LLPS [20]. As LLPS involves intrinsically disordered regions (IDRs) of proteins, we scrutinized and compared the disordered regions of HP1 proteins (Figure 1a). The C-terminal disordered region (IDR-C) was relatively conserved and only minor differences were observed at the N-terminus (IDR-N) and hinge region (IDR-H) (Figure 1a). However, HP1 homologs have different theoretical isoelectric points (pI). To investigate the HP1 proteins *in vitro*, we induced the expression of His tagged HP1s in *E. coli* and purified them using a Ni-NTA column. Purified HP1 proteins were checked by coomassie blue-stained SDS-PAGE gels (Figure S1A) and showed the expected protein sizes, HP1α (24.3 kDa), HP1β (23.7 kDa), and HP1γ (23.0 kDa). Then, we performed ion-exchange chromatography analysis and confirmed the expected pI of HP1 proteins (Figure 1b). Among the three homologs, HP1β is the most acidic protein (pI 4.85), followed by HP1γ (pI 5.13) and HP1α (pI 5.71) (Figure 1b). Further analysis revealed that this difference between HP1 homologs was most pronounced in the IDR-H. Whereas HP1α and HP1γ contain more positively than negatively charged residues in their IDR-H (15/6 and 13/8, respectively), HP1β has relatively equal numbers of positively and negatively charged residues (11/12) in the IDR-H resulting in a much lower local pI of 5.80 (Figure 1c).

### HP1β cannot self-phase separate because of its acidic linker domain

**Figure 2.**
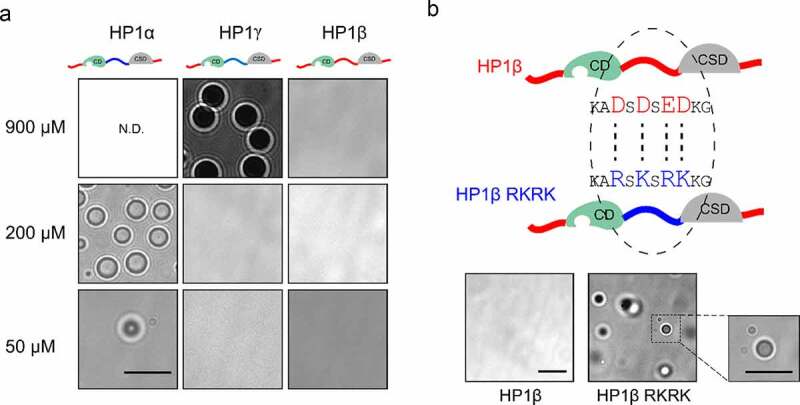
HP1β cannot self-phase separate because of its acidic linker domain. (a) DIC images of HP1 droplets at 4°C in a buffer containing 20 mM HEPES pH 7.2, 75 mM KCl and 1 mM DTT using the 63x objective of a DeltaVision Personal Microscope (scale bar: 10 µm). Protein concentrations are as indicated. N.D.: not done. (b) Phase separation of engineered HP1β at 170 µM and 4°C with four amino acid substitutions in the IDR-H changing it from acidic to basic (HP1β RKRK). A zoomed-in image is shown with the same magnification as in (a). Scale bar: 10 µm

We next systematically compared the property of HP1 homologs in phase separation. In the absence of IDR-N phosphorylation and DNA, we observed LLPS with HP1α at 200 µM and to a lesser extent at 50 µM, both at 4°C (Figure 2a). While HP1γ underwent LLPS at a higher concentration (900 µM at 4°C); HP1β did not at any of these conditions (Figure 2a). As the most distinguishing feature of HP1β is the acidic rather than basic IDR-H, we next replaced four acidic amino acids in IDR-H, including aspartic acid (D) 88, D90, glutamic acid (E) 92, and D93, with lysine (K) or arginine (R) (HP1β RKRK). Indeed, this engineered HP1β RKRK could form phase-separated droplets at concentrations as low as 170 µM at 4°C (Figures 2b and S1B) underscoring the decisive role of the basic IDR-H in LLPS. The size of the HP1β RKRK droplets was comparable with the HP1α droplets at the concentration of 200 µM (Figure 2a). As the self-phase separation of HP1 proteins is mediated by the interaction of IDR-N and IDR-H [20,22], we analyzed the correlation between the charge ratio of IDRs and HP1 protein concentration at which phase separation was observed. With a linear function fitting, we obtained an estimated concentration of HP1β self-phase separation of ~ 1.736 mM (Figure S2).

### HP1β can form phase-separated droplets in the presence of histones

**Figure 3.**
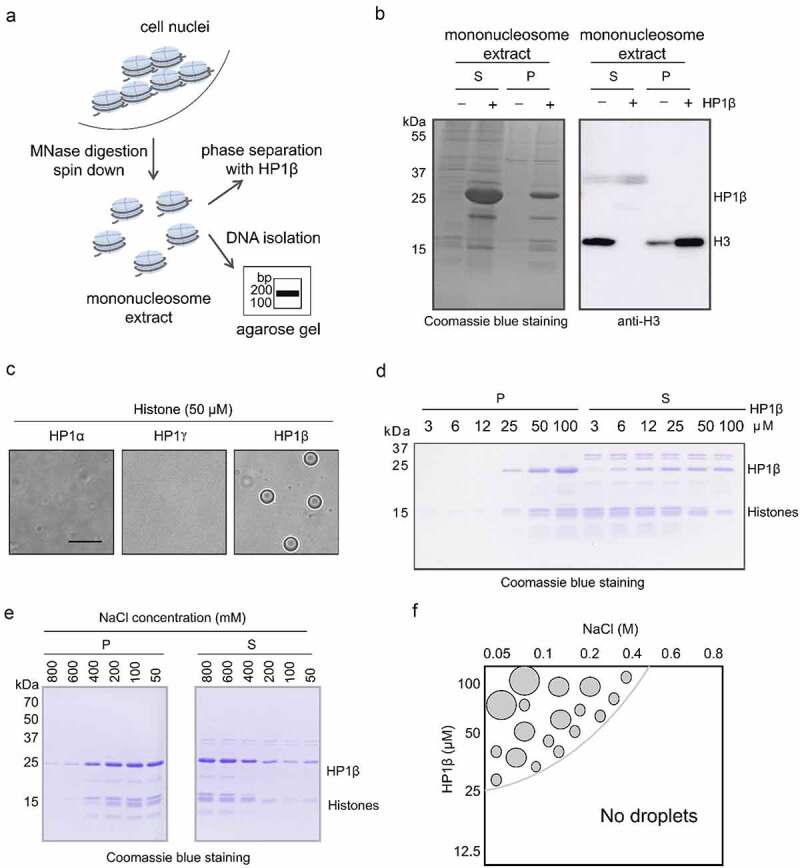
HP1β can form phase-separated droplets in the presence of histones. (a) Illustration of isolating mononucleosomes by MNase treatment (left). (b) Mononucleosome solution was incubated with or without 30 µg of HP1β at 4°C in a buffer containing 20 mM Tris-HCl pH 7.5, 150 mM NaCl, 3 mM CaCl_2_, 0.1% NP-40 and 1.5 mM EDTA. Phase-separated droplets were pelleted by centrifugation. Proteins in the supernatant (S) and phase-separated droplets (P) were separated and visualized by coomassie blue SDS-PAGE gels and western blotting with an anti-H3 antibody. (c-e) HP1 phase separation in the presence of histones isolated by acid-extraction from HEK293T cells in a buffer of 20 mM HEPES pH 7.2, 75 mM KCl and 1 mM DTT. 50 µM of HP1 homologs were incubated with 50 µM of histones (scale bar: 10 µm) (c). 3 to 100 µM of HP1β was incubated with 100 µM of histones. HP1β phase-separated droplets were separated and visualized as above (d). 50 µM of HP1β was incubated with 50 µM of histones in a buffer with NaCl concentrations ranging from 50 to 800 mM. Proteins in the P and S fractions were analyzed as above (e). (f) Phase diagram of HP1β with protein and salt concentration as order parameters. Phase separation was scored by the presence or absence of droplets in the sample

These results show that HP1β by itself hardly undergoes LLPS *in vitro*, but then again it interacts with numerous cellular proteins, which will likely affect and modulate its properties. As the most prominent known interactors are histone tails, we isolated mononucleosomes from HEK293T cells by MNase digestion (Figures 3a and S3). To isolate pure mononucleosomes, we first titrated the MNase concentration from 1.25 to 160 U/ml and used 40 U/ml for the preparation of mononucleosomes (Figure S3A and S3B). We incubated HP1β with isolated mononucleosomes, collected phase-separated droplets by centrifugation, and analyzed the precipitates by coomassie stained SDS-PAGE gel and western blot (Figures 3b and S4A). These results suggest that mononucleosomes promote HP1β phase separation as evidenced by an enrichment together with core histones in the pellet fraction.

To further examine the histone mediated phase separation, we prepared histones from human HEK293T cells by following an acid-extraction protocol [32]. We directly compared the three HP1 homologs and found that at low concentrations (50 µM) HP1α and HP1γ did not form phase-separated droplets with histones (Figure 3c). However, HP1β mixed with histones yielded an opalescent solution containing spherical droplets (Figure 3c) that fused over time, which is a central criterion for LLPS (Video S1).

Toward a mechanistic understanding of HP1β phase separation, we investigated the influence of protein and salt concentration on droplet formation in the presence of histones. To do so, we incubated different concentrations of HP1β protein (3 to 100 µM) with 100 µM of histones at 4°C in a buffer containing 20 mM HEPES pH 7.2, 75 mM KCl and 1 mM DTT. HP1β phase-separated droplets were then separated by centrifugation for visualization by coomassie stained SDS-PAGE gels (Figures 3d, S4B and S4C). As a control, we incubated BSA with histones at the same conditions and did not observe phase-separated droplets at any of the conditions (Figure S4D and S4E). However, in the presence of histones, HP1β solutions became turbid starting at concentrations as low as 25 µM, showing characteristic phase-separated droplets (Figures 3d, S4B and S4C). Droplets with 50 µM HP1β and stoichiometric amounts of histones formed up to 400 mM NaCl became smaller with increasing salt concentrations and disappeared at 600 mM NaCl (Figures 3e, S5A, S5B and summarized in Figure 3f). These results indicate that HP1β undergoes LLPS under physiological salt and protein concentrations.

### Trimethylation of K9 of histone H3 and histone dimerization are required for HP1β phase separation

Previously it was reported that linker histone H1 forms LLPS with DNA and nucleosomes [33–35]. To investigate the contribution of histone H1 to HP1β phase separation, we analyzed phase-separated droplets by western blot. We clearly detected histone H1 in the supernatants, but not in pellets of HP1β phase-separated droplets (Figure S6). This result suggests that histone H1 is not required for HP1β phase separation.

**Figure 4.**
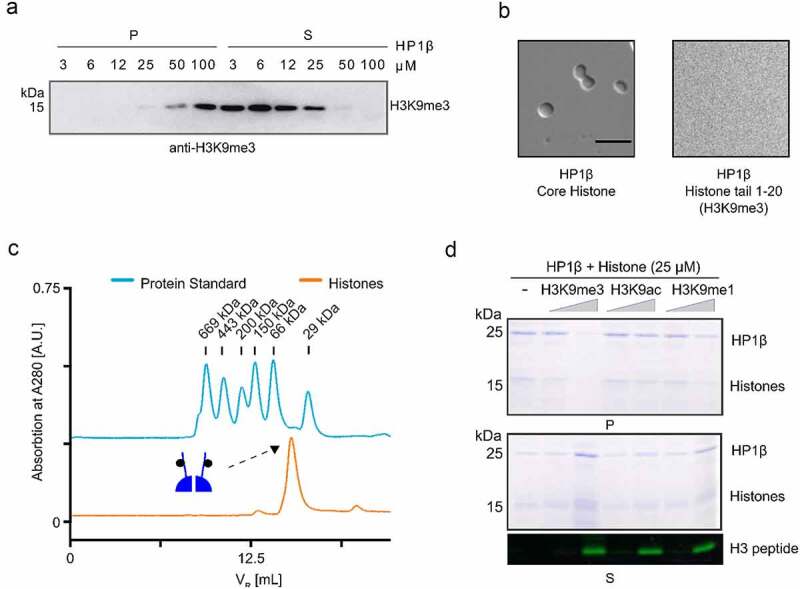
Trimethylation of K9 of histone H3 (H3K9me3) and histone dimerization are required for HP1β phase separation. (a) HP1β protein from 3 to 100 µM was incubated with 100 µM of histones at 4°C in a buffer containing 20 mM HEPES pH 7.2, 75 mM KCl and 1 mM DTT. HP1β phase-separated droplets were separated by spin down. Proteins in P and S fractions were analyzed by SDS-PAGE gels and visualized western blot with anti-H3K9me3 antibody. (b) Representative DIC images show HP1β phase separation assay outcome in the presence of histones or histone H3 peptide (aa 1–20) carrying H3K9me3. 25 µM of HP1β was incubated with either 25 µM core histones or H3K9me3 peptide (aa 1–20). (c) Analysis of histones by size exclusion chromatography (SEC). 250 µg of histones were diluted in a buffer of 20 mM Tris-HCl, 300 mM NaCl, pH 7.4 and separated on an equilibrated Superdex 200 Increase 10/300 GL column. For size comparison a protein marker mix including carbonic anhydrase (29 kDa), bovine serum albumin (66 kDa), alcohol dehydrogenase (150 kDa), beta-amylase (200 kDa), apoferritin (443 kDa), and thyroglobulin (669 kDa) was analyzed under identical conditions. (d) Histone H3 peptide (aa 1–20) carrying H3K9me3, or H3K9me1 or H3K9ac was incubated with 25 µM of HP1β and histones. Proteins in S and P fractions were analyzed and visualized by coomassie stained SDS-PAGE gels and H3 peptides by fluorescent imaging

When incubating increasing concentrations of HP1β with purified core histones, we found first histone H3 (in particular the trimethylated K9 form, H3K9me3) in droplets starting at 25 µM with a corresponding depletion from the supernatants (Figures 3d, Figures 4a and S7A), while at higher concentrations also the other core histones (H2A, H2B, and H4) were present (Figure 3d). While core histones were sufficient for HP1β LLPS, we found that H3K9me3 peptides encompassing amino acids 1–20 (aa 1–20), the binding substrate of the HP1β CSD, did not cause turbidity and droplet formation (Figure 4b). The fact that H3K9me3 histone tails were not sufficient for HP1β LLPS suggests that the remainder of the H3 histone, in particular the histone fold domains, and their ability to dimerize are required for LLPS. Indeed, size-exclusion chromatography (SEC) of histone preparations showed a major peak between 29 and 66 kDa, likely corresponding to a histone dimer (Figure 4c). We, next, performed a competition assay using H3 peptides containing either K9me3, or K9me1, or K9ac modifications added to the HP1β and histones (Figure 4d and S7B). Notably, only H3K9me3 peptides, but not H3K9me1 and H3K9ac peptides, efficiently disrupted HP1β-histone dependent LLPS.

**Figure 5.**
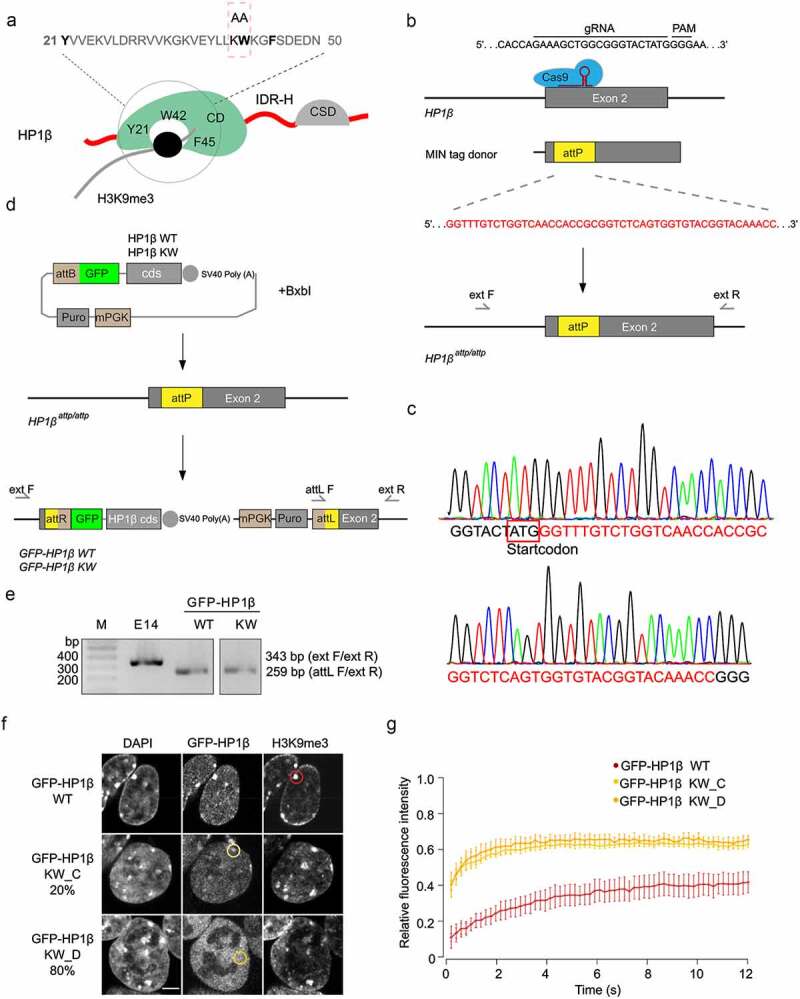
HP1β phase separation contributes to heterochromatin formation *in vivo*. (a) Illustration of the binding of H3K9me3 and the CD domain of HP1β. The amino acids, tyrosine (Y) 21, tryptophan (W) 42 and phenylalanine (F) 45, form an aromatic cage for H3K9me3 peptide that is abolished by the replacement of K41W42 with alanine (A) [15]. (b and c) Schematic representations show the CRISPR/Cas9 gene-editing strategy used to generate MIN tagged HP1β mESCs. The donor harbors the MIN tag sequence (*attP*) and homology arms to the genomic sequence 5ʹ and 3ʹ of the translational start site. The targeting region was amplified with primers as indicated and assessed by Sanger sequencing. (d) Schematic representation shows the strategy to generate GFP-HP1β WT and KW mESC lines with Bxb1 mediated recombination. (e) Gel electrophoresis of the multiplex PCR for validation of GFP-HP1β mESCs with primers as indicated in (d). 343 bp and 259 bp sequences were amplified from E14 and GFP-HP1β cells, respectively. (f) Representative images of GFP-HP1β WT and KW mESCs stained with an anti-H3K9me3 antibody. Scale bar: 5 µm. See overview images in Figure S10. (g) FRAP quantification of GFP-HP1β WT and GFP-HP1β KW. Curves show average GFP signal relative to the fluorescence signal prior to bleaching (WT, n = 20 and KW_C (chromocenter), n = 6 and KW_D (diffuse), n = 6). The areas used for FRAP are indicated by circles in (f)

The amino acids in the CD domain, including tyrosine (Y) 21, tryptophan (W) 42, and phenylalanine (F) 45, form an aromatic cage for H3K9me3 binding (Figure 5a). The replacement of K41 and W42 with alanine (HP1β KW) is sufficient to abolish the H3K9me3 binding of HP1β [15,16]. We purified HP1β KW and incubated different concentrations of the mutant proteins (6 to 25 µM) with 25 µM histones. By analyzing coomassie stained SDS-PAGE gels, we found that almost half of histone H3 was still detected in the supernatant of phase-separated droplets of HP1β KW at the concentration (12 µM), while H3 was nearly completely depleted from the supernatant into the pellet of HP1β WT droplets (Figure S8A and S8B). This concentration corresponds to the physiological HP1β concentration measured at heterochromatin [36]. At the higher concentration (25 µM), HP1β KW formed phase-separated droplets similar to HP1β WT, which may be due to the unspecific binding with histones (Figure S8A and S8B). These results indicate that HP1β KW, which is deficient in binding H3K9me3, is less efficient in forming phase-separated droplets at physiological concentrations.

To study the function of HP1β phase separation *in vivo*, we generated a mouse embryonic stem cell (mESC) line carrying the GFP-HP1β KW mutant as well as a wild type using the MIN tag genome engineering strategy, called MINtool [31]. The MINtool allows to replace the endogenous gene of interest with the mini gene products that carry mutations or tags. With this strategy, a multifunctional integrase (MIN) tag sequence was first inserted into the open reading frame of *HP1β* directly downstream of the start codon by the CRISPR/Cas9 genome editing tool (Figure 5b and c). By Bxb1-mediated recombination, the coding sequences for GFP-HP1β WT and GFP-HP1β KW were subsequently integrated into the locus (Figure 5d). With specific primers, 343 bp and 259 bp sequences were amplified from the MIN tagged and GFP tagged HP1β cell lines, respectively (Figure 5e). We performed western blot analysis and found that the levels of GFP-HP1β WT and KW in the engineered cells are higher than the endogenous HP1β levels in WT mESCs (Figure S9). In line with previous publications, GFP-HP1β WT is predominantly localized at the chromocenters (Figure 5f). GFP-HP1β KW, on the other hand, showed a dispersed nuclear distribution (KW_D) in 80% of the mutant cells with no accumulation at heterochromatin compartments, while it slightly accumulated at the chromocenters (KW_C) in 20% of the cells (Figures 5f and S10). To measure the kinetics of binding in living cells, fluorescence recovery after photobleaching (FRAP) analyses were performed and evaluated. These showed a similar kinetics of recovery of GFP-HP1β KW_C and KW_D that is substantially faster than GFP-HP1β WT (Figure 5g).

**Figure 6.**
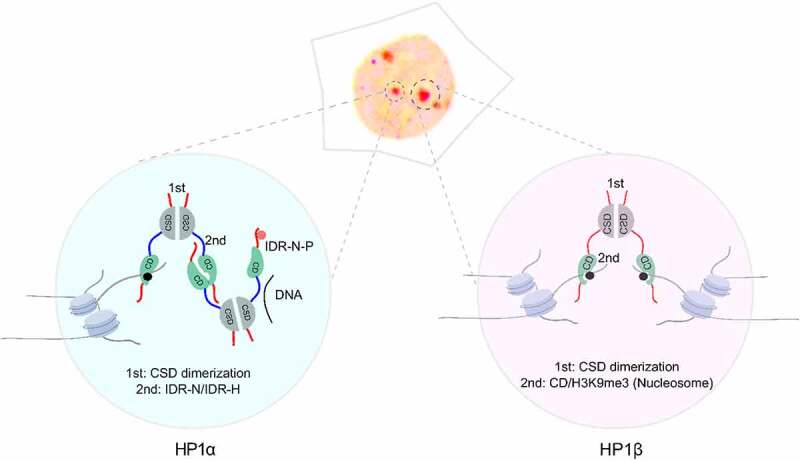
Model of HP1α/γ and HP1β phase separation contributing to heterochromatin formation *in vivo*. The interaction of IDR-N and IDR-H is an essential valency for HP1α and HP1γ phase separation (left). Although HP1α and HP1γ contain basic IDR-H, the minor difference leads to a threshold phase separation concentration higher than the physiological concentration for HP1γ. The negatively charged DNA and phosphorylation (P) of IDR-N can promote HP1α phase separation. In contrast, HP1β phase separation is more complex and requires the CSD mediated dimerization and the binding of the CD domain to the H3K9me3 nucleosome (right)

Altogether, our results show that all three HP1 proteins can in principle form phase-separated droplets *in vitro* but require different conditions. While LLPS of HP1α/HP1γ mostly relies on the interaction of IDR-N and IDR-H (Figure 6), HP1β phase separation requires the binding of H3K9me3 nucleosomes (Figure 6). These multivalent interactions are required for the formation of oligomeric structures and phase-separated droplets *in vitro*. HP1β dimerization and binding of two H3K9me3 histone tails thus contribute to heterochromatin clustering *in vivo*.

## Discussion

The three HP1 homologs are considered important regulators of heterochromatin formation and spreading. HP1α, but not HP1β and HP1γ, was shown to form LLPS driving heterochromatin formation [20,22], raising the question of which molecular determinants are responsible for these differences. A comparison shows that all three HP1s share a common overall structure but differ in the net charge of their IDR-H (Figure 1). We found that HP1γ, similar to HP1α, contains a basic IDR-H and indeed forms phase-separated droplets albeit at high concentrations of about 0.9 mM, which is, however, four times higher than the 0.2 mM used for HP1α and way beyond the reported physiological concentrations of about 10 µM [36,37]. Here, we showed that HP1β has a slightly acidic IDR-H in contrast to the very basic one of HP1α and HP1γ. Our further finding that HP1β WT does not form phase-separated droplets, but could be engineered to do so simply by changing four acidic to basic amino acids in the IDR-H, supports the notions that HP1α (and HP1γ) LLPS relies on interactions between their acidic IDR-N and their basic IDR-H. Previously, it was shown that the addition of negative-charged DNA promotes the phase separation of HP1α but not of HP1β [20,24]. Considering the difference of HP1α and HP1β IDR-H regions, we added positive-charged histones and found that HP1β showed phase-separated droplets even at concentrations as low as 25 µM. In line with our findings, it was shown that HP1β together with SUV39H1 forms phase-separated droplets in the presence of nuclear extracts [38]. The mode of HP1β LLPS differs and requires the binding of H3K9me3 nucleosomes (Figure 6). Interestingly, HP1α and HP1γ do not phase separate under these conditions, although they have functionally similar CD domains binding H3K9me3 and a CSD for dimerization. We speculate that the interaction of their acidic IDR-N and basic IDR-H antagonizes oligomerization via histone H3K9me3 binding [39]. In any case, our study identified the net charge of the IDR-H as a critical feature controlling LLPS of HP1 *in vitro*. The observation that the simple addition of histones promotes LLPS with HP1β indicates that the situation *in vivo*, with its numerous direct and indirect interactions, is much more complex.

As diverse as the phase separating properties of HP1s are *in vitro*, so are their subcellular distribution and function *in vivo*. While HP1γ is predominantly localized in euchromatin, HP1α and HP1β are mostly associated with heterochromatin [40]. Whereas HP1α plays a central role in the formation of satellite heterochromatin, HP1β is involved in chromocenter formation by bridging H3K9me3 containing nucleosomes [29,41–43]. Interestingly, histone acetylation was recently described to drive LLPS and chromatin organization [33]. These results suggest that histone tail modifications in combination with specific reader proteins may encode the establishment of functionally distinct chromatin domains in the nucleus. The recent observation of HP1 independent formation of heterochromatin in cultured cell lines [24] serves as a reminder that there are several mechanisms that may cooperate or compete in the establishment of heterochromatin states in vivo. Future comprehensive studies are needed to dissect their relative contributions in different cell types throughout differentiation.

## Supplementary Material

Supplemental Material
