# Supplementary material for: HP1β carries an acidic linker domain and requires H3K9me3 for phase separation
Source: Nucleus. 2021 Mar 4;12(1):44–57. doi: 10.1080/19491034.2021.1889858 (PMC7939559; doi:10.1080/19491034.2021.1889858)
Supplement: Supplemental Material [file KNCL_A_1889858_SM1369.zip › Supporting information_revised.pdf]

Supporting Information

HP1β carries an acidic linker domain and requires H3K9me3 for phase separation

Weihua Qin[1*], Andreas Stengl[1], Enes Ugur[1,2], Susanne Leidescher[1], Joel Ryan[1], M. Cristina Cardoso[3], Heinrich Leonhardt[1*]

Supporting information includes 10 supplementary Figures and 1 supplementary video.

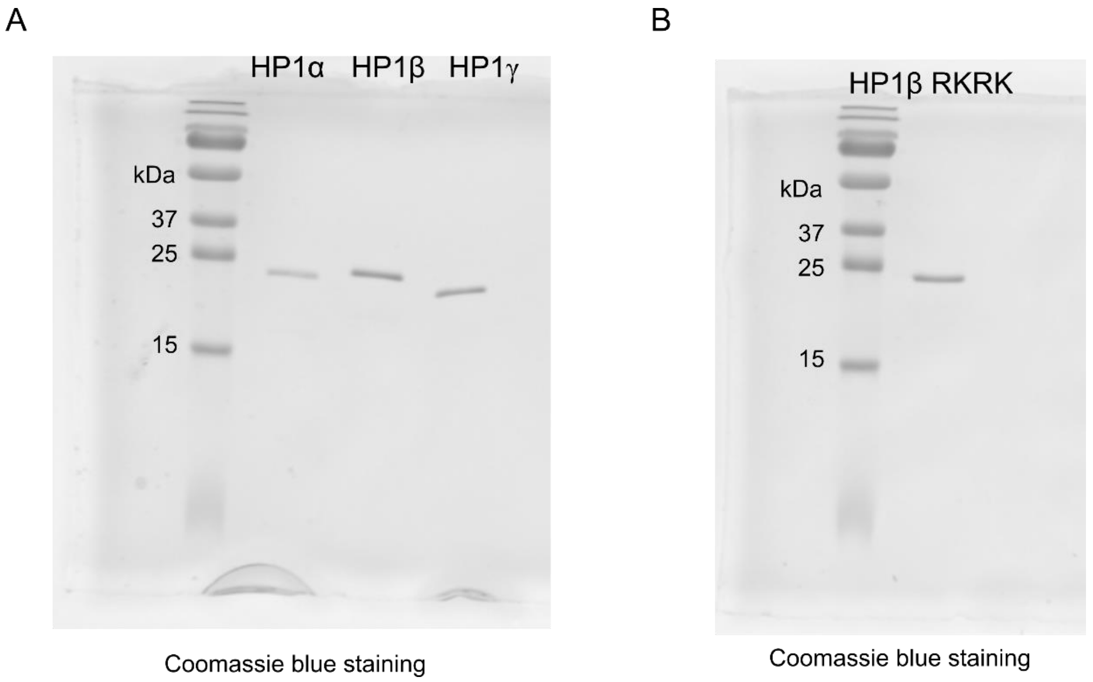

Figure S1. Purification of recombinant HP1 proteins produced in *E. coli*. His-tagged HP1α, β, γ (A) and HP1β RKRK (B) proteins were purified using a Ni-NTA affinity column. 500 ng of purified proteins were analyzed by SDS-PAGE and visualized by coomassie blue staining. The expected protein sizes were calculated using a website tool (https://web.expasy.org/compute_pi/), 6xHis-HP1α: 24.3, 6xHis-HP1β: 23.7, 6xHis-HP1γ: 23.0 and 6xHis-HP1β RKRK: 23.8 kDa.

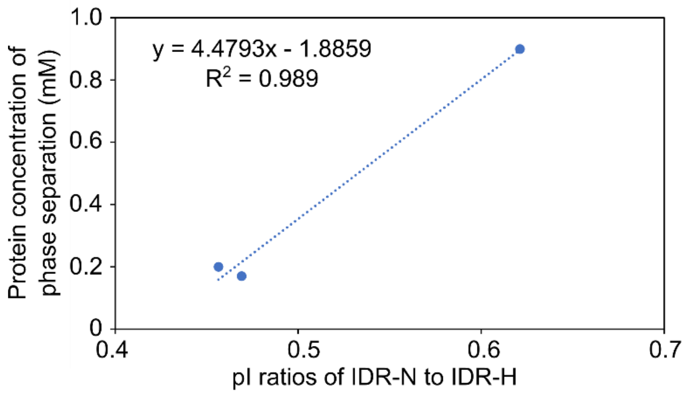

	pI ratio of IDR-N to H	Concentration of phase separation (mM)
HP1β RKRK	0.469 (4.69/10)	0.170
HP1α	0.46 (4.66/10.21)	0.200
HP1γ	0.62 (6.1/9.82)	0.900

	pI ratio of IDR-N to H	Concentration of phase separation (mM)
HP1β	0.81 (4.69/5.8)	1.736 (N.D)
Calculation by linear function fitting	$y = 4.4793x - 1.8859$ $R^2 = 0.989$	

Figure S2. Linear plots show the correlation of HP1 pI and phase separation. A linear plot is generated by the charge ratio of IDR-N to IDR-H and the concentration of HP1 proteins observed. Based on the linear fitting equation, the estimated self-phase separation of HP1β was calculated and shown below.

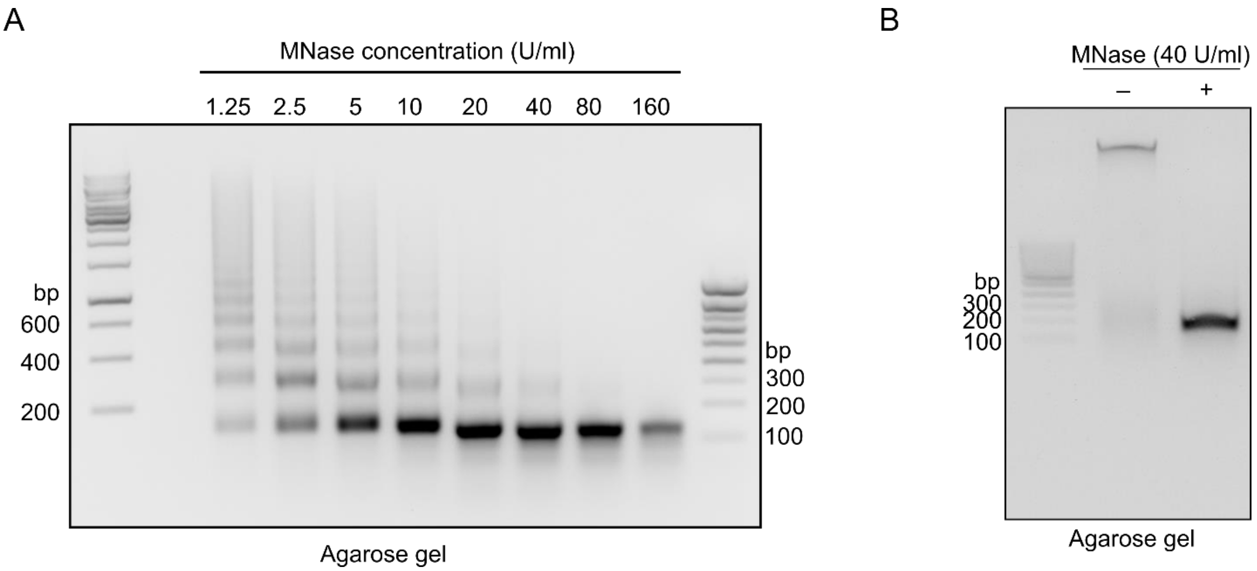

Figure S3. Validation of mononucleosomes isolated by MNase digestion by analysis in agarose gels. 1.5 µg DNA extracted from cells treated with different amounts of MNase at 37 °C for 5 min were analyzed in 1.5% agarose gels (A). Validation of mononucleosomes used in Figure 3B (B).

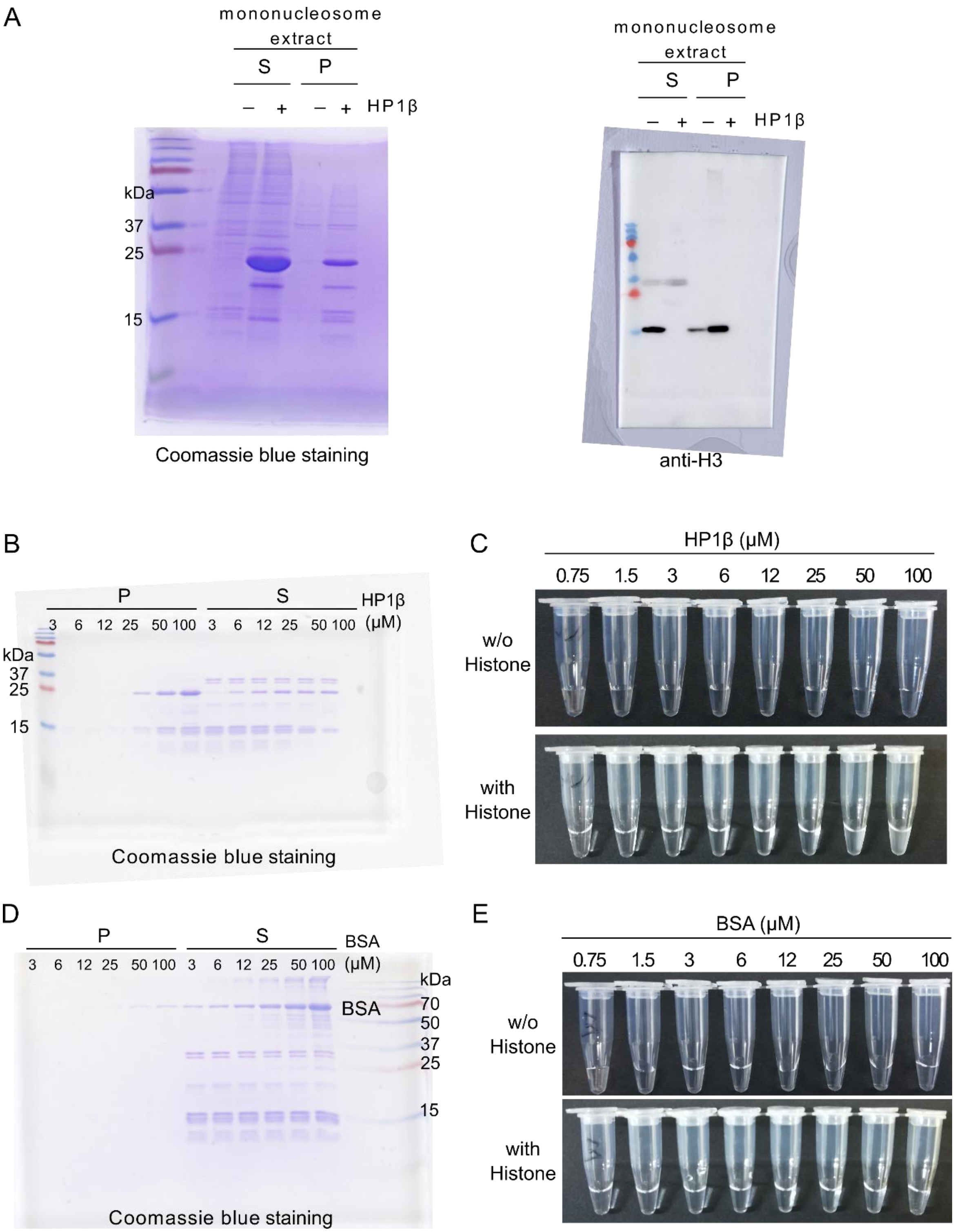

Figure S4. (A) Original coomassie and western blot gel corresponding to Figure 3B. (B) Original coomassie gel corresponding to Figure 3D, for details see Figure 3. (C-E) Protein concentration dependent phase separation of HP1β or BSA as control in B/C and D/E, respectively. HP1β and BSA from 0.75 to 100 μM concentration was incubated with or without 100 μM of histones as indicated in a buffer containing 20 mM HEPES pH 7.2, 75 mM KCl and 1 mM DTT at 4 °C for 5 min.

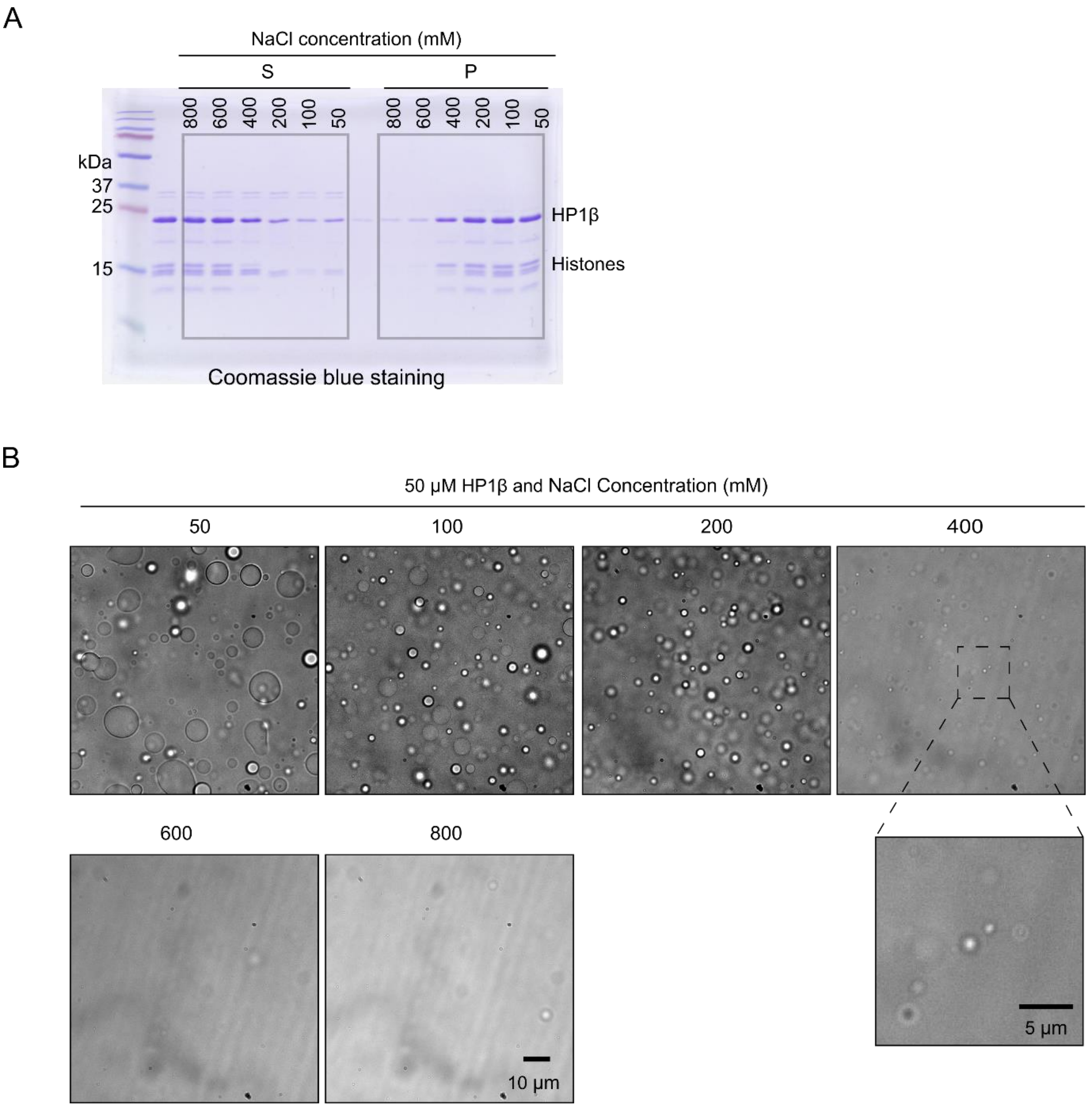

Figure S5. Salt concentration dependent phase separation of HP1β. (A) Original coomassie gel corresponding to Figure 3E. (B) 50 μM of HP1β was incubated with 50 μM of histones in a buffer containing 10 mM Tris-HCl pH 7.5 and 1 mM DTT with NaCl concentrations ranging from 50 to 800 mM. The phase-separated droplets were visualized using the 63× objective of a DeltaVision Personal Microscopy (scale bar: 10 μm and 5 μm, as indicated). For details see Figure 3.

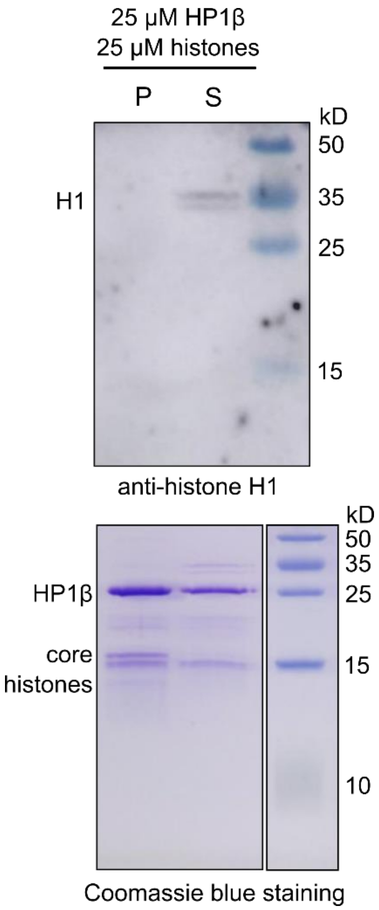

Figure S6. Analysis of histone H1 upon HP1β phase-separation. 25 μM HP1β was incubated with 25 μM histones in a buffer of 20 mM HEPES pH 7.2, 75 mM KCl and 1 mM DTT. Proteins in the supernatant (S) and pellets (P) were separated in SDS-PAGE and detected by both an anti-H1 antibody and a coomassie blue stained gel.

A

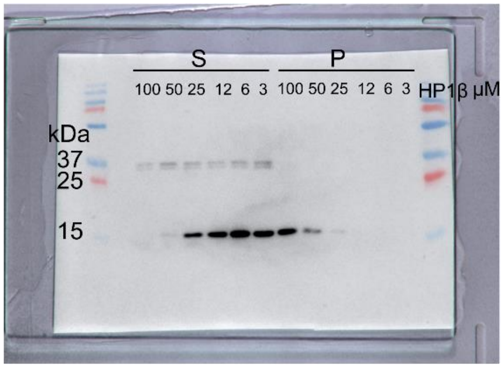

anti-H3K9me3

B

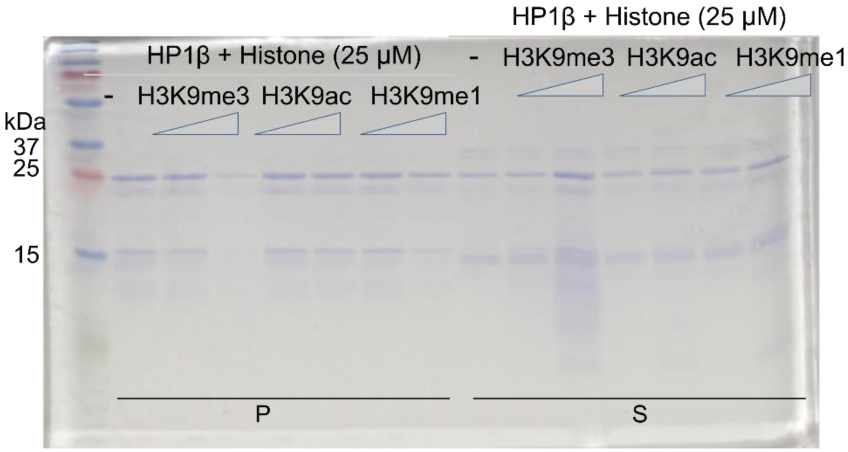

Coomassie blue staining

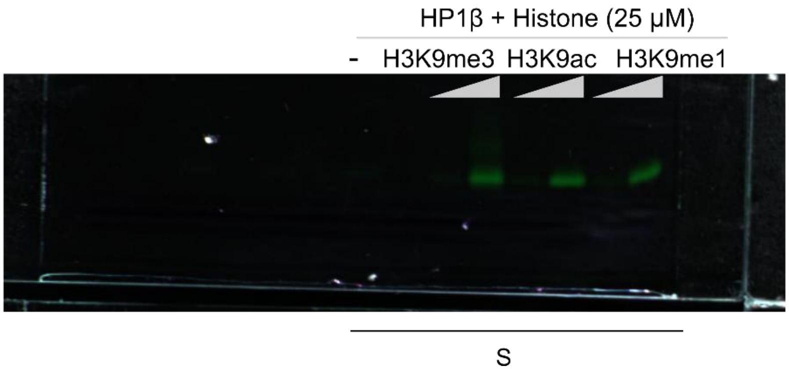

Figure S7. Original western blot and coomassie stained gels corresponding to Figure 4A and 4D. For details see Figure 4.

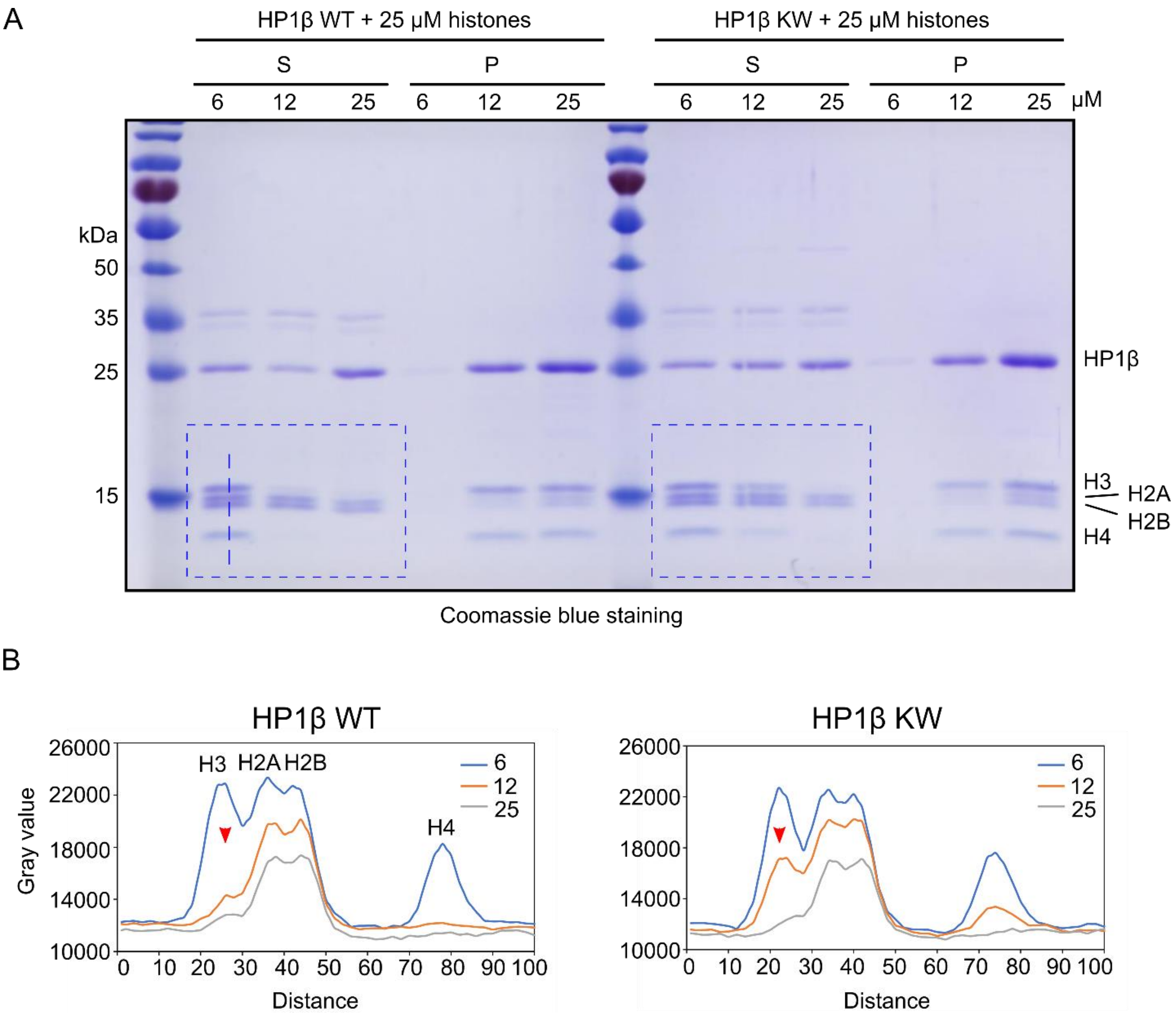

Figure S8. Phase separation of HP1β KW in the presence of histones. Different concentrations of HP1β WT and KW mutant proteins (6 to 25 μM) were incubated with 25 μM histones at 4 °C in a buffer of 20 mM HEPES pH 7.2, 75 mM KCl and 1 mM DTT. Phase-separated droplets were pelleted by centrifugation. Proteins in the S and P were separated and visualized by coomassie blue SDS-PAGE gels (A). (B) Line scans along the core histones in the S of HP1β WT and KW droplets. The H3 peak in the S at the concentration of 12 μM was highlighted with red arrowheads.

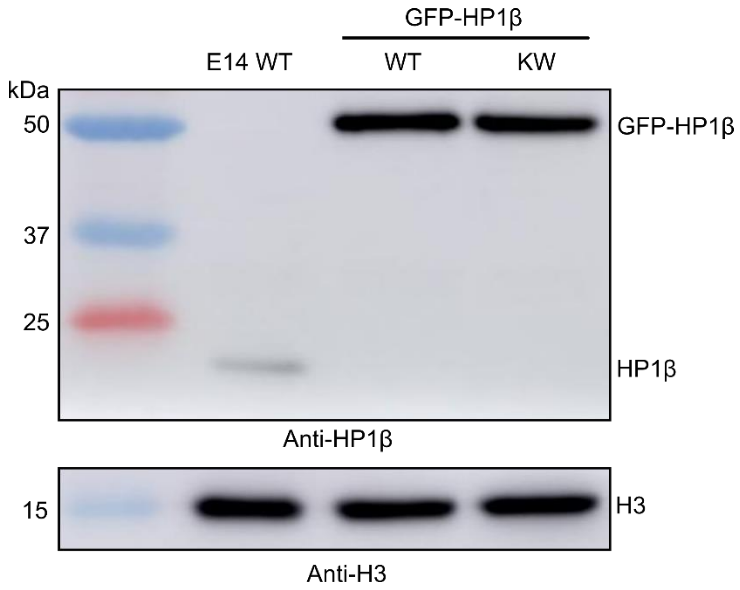

Figure S9. Characterization of GFP-HP1β WT and GFP-HP1β KW mESCs by western blot using an anti-HP1β antibody. The anti-H3 western blot was used as a loading control.

A

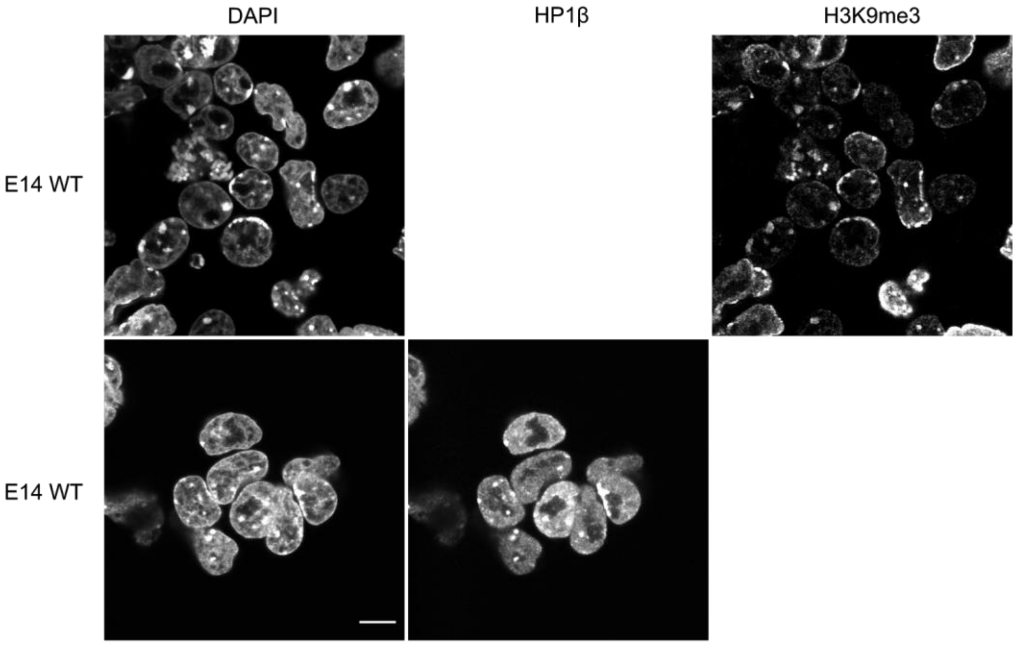

B

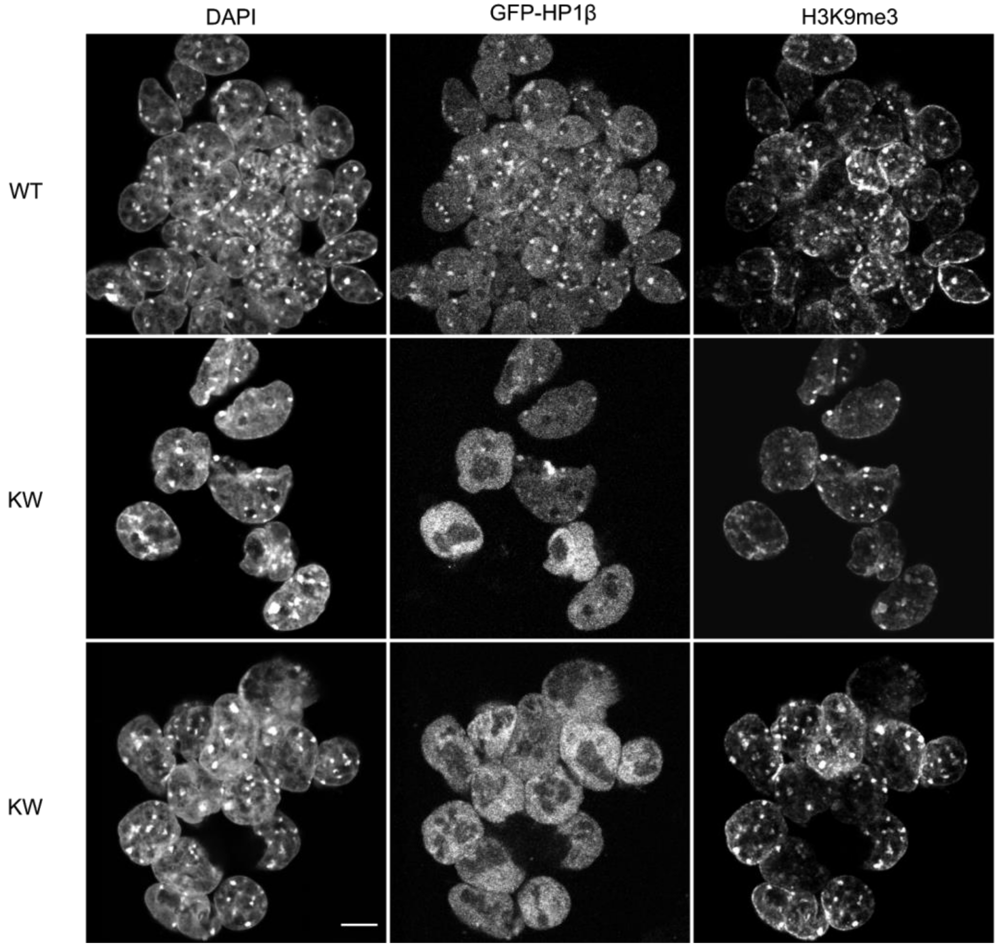

Figure S10. Representative images of E14 WT mESCs stained with anti-HP1β or anti-H3K9me3 antibodies (A) and GFP-HP1β WT and KW mESCs stained with an anti-H3K9me3 antibody (B). Scale bar: 10 μm.

Video S1. HP1β phase separation with histones. 50 μM of HP1β was incubated with 50 μM of histones in a buffer containing 10 mM Tris-HCl pH 7.5, 75 mM KCl and 1 mM DTT. Phase-separated droplets were visualized using the 63× objective of a DeltaVision Personal Microscope. Scale bar: 5 μm.
